# The Use of Chemoprophylaxis after Floods to Reduce the Occurrence and Impact of Leptospirosis Outbreaks

**DOI:** 10.3390/ijerph14060594

**Published:** 2017-06-03

**Authors:** Maria Cristina Schneider, Jorge Velasco-Hernandez, Kyung-duk Min, Deise Galan Leonel, David Baca-Carrasco, Matthew E. Gompper, Rudy Hartskeerl, Claudia Munoz-Zanzi

**Affiliations:** 1 PAHO Health Emergencies Department, Pan American Health Organization, Washington, DC 20037, USA; minkyu@paho.org (K.-d.M.); galandei@paho.org (D.G.L.); 2 Instituto de Matematicas, National Autonomous University of Mexico, Juriquilla 76230, Mexico; jx.velasco@im.unam.mx (J.V.-H.); dbc270582@gmail.com (D.B.-C.); 3 School of Natural Resources, University of Missouri, Columbia, MO 65211, USA; gompperm@missouri.edu; 4 WHO/FAO/OIE and National Leptospirosis Reference Centre, Amsterdam 1105, The Netherlands; rudyhartskeerl@gmail.com; 5 Division of Epidemiology and Community Health, School of Public Health, University of Minnesota, Minneapolis, MN 55455, USA; munozzan@umn.edu

**Keywords:** leptospirosis, chemoprophylaxis, extreme weather, outbreaks

## Abstract

Record-breaking and devastating rainfall events have occurred in the past decade. Rain and floods are considered the main risk factors for leptospirosis and several outbreaks have been reported following extreme weather events. In such situations, one possible intervention to prevent leptospirosis cases in high-risk groups is the use of chemoprophylaxis. However, not enough evidence of its effect is available. The objectives of this study were to review the literature on the current practices of chemoprophylaxis for leptospirosis and to explore, using a mathematical model, how various chemoprophylaxis scenarios may affect the progression of a leptospirosis outbreak. Twenty-six peer-reviewed publications were selected (10 quantitative studies, two systematic reviews and 14 articles of other types). Oral doxycycline was the most used antibiotic for chemoprophylaxis of leptospirosis. Post-exposure prophylaxis was assessed in four studies following a natural disaster. Although evidence of the effectiveness of post-exposure prophylaxis is inconsistent, the direction of association supported a protective effect for morbidity and mortality. The theoretical model showed how the assumed benefit of chemoprophylaxis was influenced by the time and rate of administration. Future models should consider the heterogeneity of affected communities, improved estimates of the effect of chemoprophylaxis on leptospirosis infection and disease, as well as potential detrimental impacts. Additional research is critical to provide clear evidence-based recommendations for leptospirosis control during an outbreak. The results of this study suggest that chemoprophylaxis may provide some protection in reducing the number of leptospirosis cases after a high-risk exposure; however, the effective benefit may depend on a variety of factors such as the timing and coverage of prophylaxis. The information summarized can be used to support decision-making during a high-risk event.

## 1. Introduction

Over the past 60 years, extreme weather and climatic events have been observed across the globe [1,2]. Within the past decade, record-breaking and devastating rainfall events have occurred, and 2010 was ranked the wettest year on record [3,4]. Rain and floods are considered primary risk factors for leptospirosis and outbreaks have been reported around the world following extreme weather events in diverse locales such as Brazil, Guyana, Italy, New Caledonia, Nicaragua, the Philippines, and the United States [5,6,7,8,9,10,11,12,13]. Due to climate change, the frequency and intensity of extreme weather events, especially heavy rainfall and floods are projected to increase [14,15]. Consequently, the risk of leptospirosis, which is a primary communicable disease of concern associated with such natural disasters [16], has been exacerbated.

The risks associated with leptospirosis outbreaks in the Americas Region (Latin America and the Caribbean) may be particularly high. Annually, approximately ten million people are affected by natural disasters in the Americas Region, with the majority of them corresponding to storms (41%), and floods (35%) [17,18,19]. The most affected sub-regions are Central America and the Caribbean [17]. Analyzing the International Disaster Database from the Centre for Research on the Epidemiology of Disasters (CRED), there were 356 natural disasters in Central America between 2000 and 2016 [2]. Of these, 136 were floods (including 108 riverine floods) and 109 were storms (including 103 tropical cyclones), equating to 68.8% of the total natural disasters in the period [2] (Appendix Table A1).

Leptospirosis is one of the most widespread zoonotic diseases worldwide, usually transmitted through contact with an environment contaminated with the urine of an infected animal [20,21]. The greatest burden of disease affects resource-poor populations in tropical regions, mainly in developing countries [22,23,24]. A recently published systematic review estimated that leptospirosis causes 1.03 million cases and 58,900 deaths each year, placing the disease among the leading zoonotic causes of morbidity and mortality around the world [22]. In the Region of the Americas, a total of 10,702 human cases were officially reported in 2014 [25]. Since the 1990s, several countries in Central America have had a history of leptospirosis outbreaks [11,26,27,28,29,30] and have being developing activities to prevent and control the disease. The Central America Isthmus includes seven countries (population ranging from 400,000 to 16 million people), with middle to low gross national income per capita and with several countries presenting approximately 40% of their population living in rural areas [31]. Studies in Central America have identified environmental and socioeconomic factors that increase the risk of leptospirosis, such as volcanic origin soil, precipitation, higher percentage of rural population, greater unsatisfied basic needs for improved housing and sanitary services, as well as extreme poverty and higher illiteracy rates [32,33]. Furthermore, the number of leptospirosis cases increases considerably during the months of heavy rain along the Pacific coast, which is when floods and hurricanes usually occur [33].

**Figure A1:**
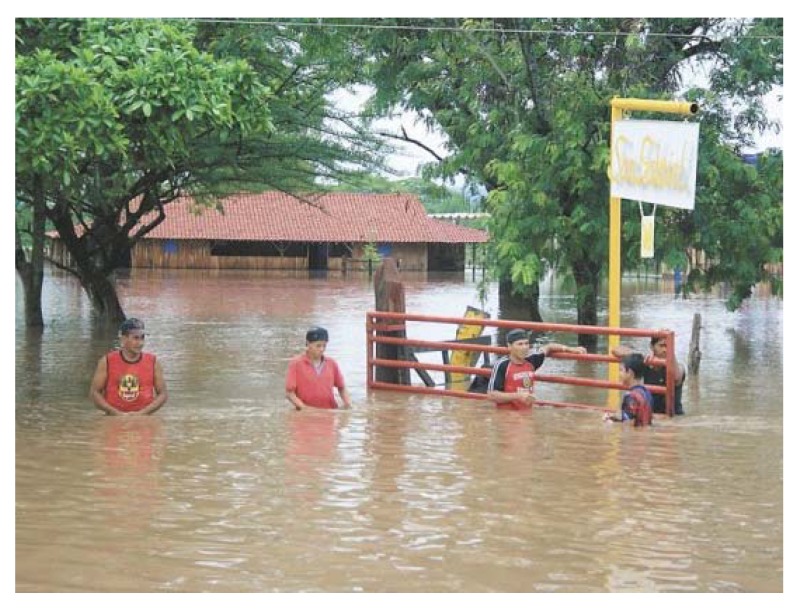
Flood in the Department of Leon Nicaragua (photo credit: Gilberto Moreno).

**Figure A2:**
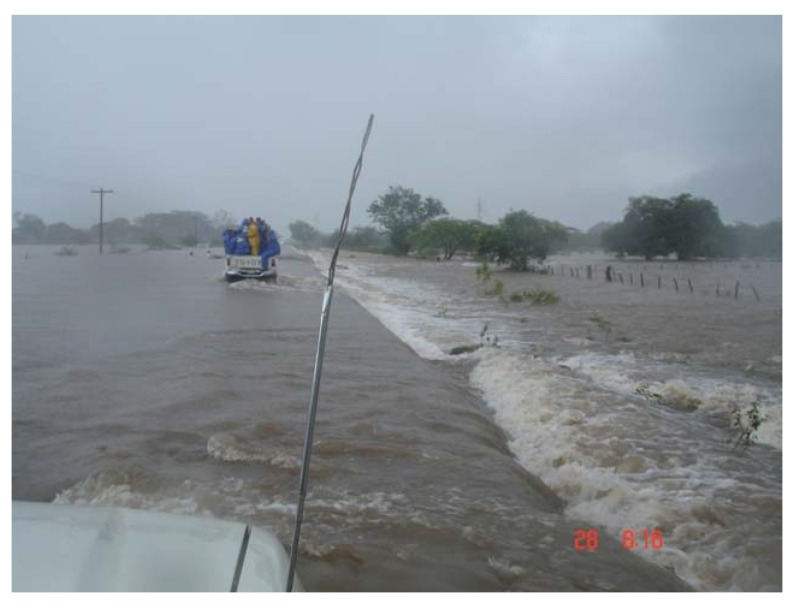
Road after a flood in Leon, Nicaragua (photo credit: Gilberto Moreno).

**Figure A3:**
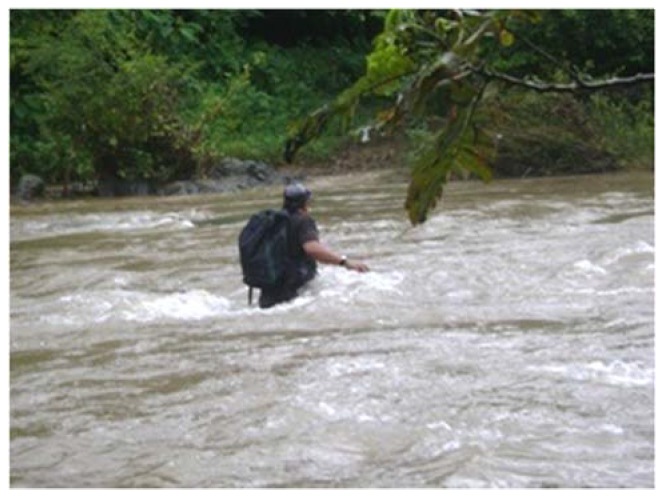
Health workers after a flood bringing health care to an affected population in Achuapa, Nicaragua (photo credit: Gilberto Moreno).

Early diagnosis and treatment of leptospirosis clinical cases is recommended for better prognosis and lower fatality, which can reach 5% to 15% [23]. However, many times after flood events roads are blocked, making the access to health facilities difficult or impossible. In addition, health systems and services may be disrupted, leaving many without access to health care [34] (Appendix Figure A1, Figure A2 and Figure A3). One possible intervention for high-risk groups mentioned in the literature is the use of population-scale chemoprophylaxis, which has been used when exposure is known to have occurred, especially in high-risk environments [20]. Mass chemoprophylaxis has been used after leptospirosis outbreaks following floods and or during water-sports events, and in Latin America it has been used as a preventive measure in high-risk situations and when access to the health facilities was difficult [35,36,37,38]. However, there is insufficient evidence to determine if such mass chemoprophylaxis should be recommended after natural disasters that result in floods in areas where leptospirosis may be a disease of high-risk outbreak potential. A systematic review published in 2009 assessed the evidence of antibiotic prophylactic use against leptospirosis and concluded that the benefits of chemoprophylaxis were unclear [39].

Given the current use and associated unclear outcomes of mass chemoprophylaxis to reduce leptospirosis health impact, the primary objective of this study was to undertake a review of the literature on the current practices and to summarize the reported outcomes. Secondly, we use a mathematical modeling approach to assess how various chemoprophylaxis scenarios may affect the progression of a leptospirosis outbreak in a population. Both objectives are conducted with the aim of supporting decision making.

## 2. Materials and Methods

**Figure 1:**
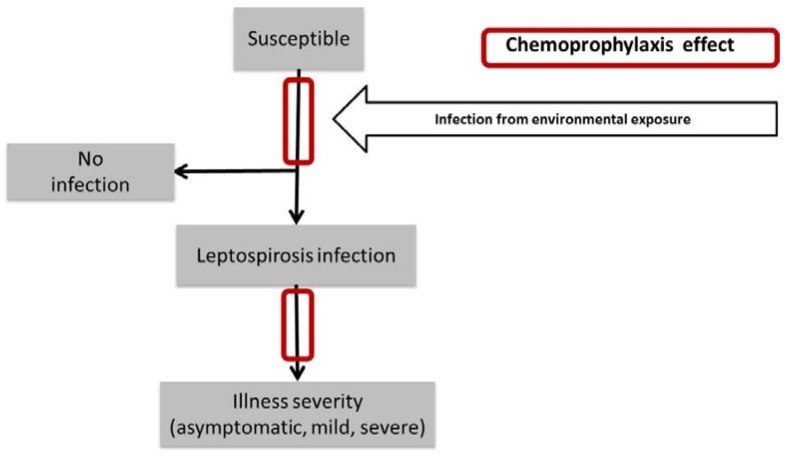
Conceptual diagram of the potential effect of chemoprophylaxis on the dynamics of a leptospirosis outbreak and its health impact.

In the first part of this study, a literature review was conducted on mass chemoprophylaxis use for leptospirosis, including antibiotic type and administration schedules. In the second part, a basic mathematical model was created to simulate a leptospirosis outbreak after a flood event (Figure 1). For the model, different scenarios for timing and uptake of chemoprophylaxis were used. The main outcome of interest was reduction in leptospirosis cases.

### 2.1. Literature Review on Chemoprophylaxis Use for Leptospirosis

To review the antibiotic regimens of chemoprophylaxis use for leptospirosis and evaluate their reported effectiveness, especially in flood-related settings, a literature review was performed. We applied extensive search strategies with two core databases: MEDLINE and Embase, without a date restriction. The Medical Subject Heading (MeSH) or “All Fields” search terms used were: (“leptospirosis” [MeSH] OR “leptospirosis” [All Fields]) AND (“chemoprevention” [MeSH] OR “chemoprevention” [All Fields] OR “chemoprophylaxis” [All Fields]) AND (“humans” [MeSH] OR “humans” [All Fields] OR “human” [All Fields]). A snowball approach was also employed from original and review articles. Snowballing refers to reviewing papers of interest and using the references in, or citations to, the paper to identify additional relevant papers [40]. Only publications in English were included in our analysis. Studies from the peer-reviewed literature, including observational and experimental studies, systematic reviews, case reports and review articles were identified. Animal studies or grey literature were excluded. The list of other publications found during the search on the effectiveness of chemoprophylaxis for leptospirosis is included in Appendix B.

The final list of articles were categorized as: (1) quantitative studies and systematic reviews; (2) others, more descriptive articles (non-systematic reviews, case reports, clinical recommendations, among others). Information was extracted from each study by reviewing the full text. Types of studies, sample size, suggested antibiotic regimens and their effectiveness were extracted from quantitative studies and systematic reviews, and recommended antibiotic regimens and descriptions of their effectiveness were recorded from other articles.

### 2.2. The Mathematical Model

The model for human leptospirosis is a compartmental SEIR model where the initials stand for susceptible (S), latently infected (E), infectious (I), and recovered or immune (R). A stage (Q) was allowed to indicate transition to being protected under chemoprophylaxis for people in S, E and I. As an environmentally-driven infection in humans, the infection from the environment takes place at a rate *f* that depends on the abundance of bacteria present in the environment. The function *f* = *βB*/(*k* + *B*) has two parameters and assumes a Michaelis-Menten form where *β* is the per capita infection rate (1/day) produced by a unit of concentration of bacteria and *k* is the half saturation bacterial concentration [41]. The compartment *B* represents the environment and it is assumed to receive leptospires shed from animals outside this explicit system at a rate of h. Leptospires from infected people contributes to *B* at a negligible rate (0.001) represented by h; however, it is kept in the equation to allow the computational approximation of the basic reproduction number (R_0_). Leptospires in *B* are depleted at a rate d. People move from E to I at rate of 1/*γ*, the incubation period, and from I to R at a rate of 1/*η*, the infectious period. Because we considered a short outbreak period (at most 30 days), birth and mortality rate were taken both to be *µ* = 0, such that no births or deaths due to other causes occurred during the outbreak. *Leptospira*-infected individuals are distributed according to the possible course of the clinical disease as severe, mild or asymptomatic which were represented by I_S_, I_M_, or I_A_, respectively. The distributions for severe, mild, and asymptomatic were assigned with probabilities *p*, *q* and 1 − *p − q,* respectively.

Chemoprophylaxis is applied at a rate *θ* to individuals who are in any of the disease compartments (S, E, I, R) at the time of administration. Individuals in R are not affected by the application since they have already recovered from the infection and have become immune. All other individuals go to the Q compartment which determines the effect of chemoprophylaxis for a given leptospirosis infection status. Specifically, while in Q, susceptible individuals (S) do not get infected and illness severity distribution can be modified following Q. The probability that an individual leaves Q as susceptible is *q_1_* and as infected is (1 − *q_1_*), with probability of illness severity determined by *p_1_*, *p_2_* and 1 − *p_1_* − *p_2_* for severe, mild, and asymptomatic, respectively. It is assumed that individuals last an average of 1/*φ* days under the action of the chemoprophylaxis. Individuals who are moved from E to Q, cannot return to E since the latency period duration, 1/*γ*, is considered to be shorter that the duration of the average residence in Q. The equations incorporated in the model are presented below:

(1) $S^{\prime }=\mu -fS-\left(\mu +\theta \right)S+q_{1}\varphi Q$
(2) $E_{S}^{\prime }=pfS-\left(\mu +\;\gamma +\;\theta \right)E_{S}$
(3) $E_{M}^{\prime }=qfS-\left(\mu +\;\gamma +\;\theta \right)E_{M}$
(4) $E_{A}^{\prime }=\left(1-p-q\right)fS-\left(\mu +\;\gamma +\;\theta \right)E_{A}$
(5) $I_{S}^{\prime }=\gamma E_{S}-\left(\mu +\eta +\theta \right)I_{S}+p_{1}\left(1-q_{1}\right)\varphi Q$
(6) $I_{M}^{\prime }=\gamma E_{M}-\left(\mu +\eta +\theta \right)I_{M}+p_{2}\left(1-q_{1}\right)\varphi Q$
(7) $I_{A}^{\prime }=\gamma E_{A}-\left(\mu +\eta +\theta \right)I_{A}+\left(1-p_{1}-p_{2}\right)\left(1-q_{1}\right)\varphi Q$
(8) $R^{\prime }=\eta \left(I_{s}+I_{M}+I_{A}\right)-\mu R$
(9) $B^{\prime }=h+h^{0}\left(I_{S}+I_{M}+I_{A}\right)-dB$
(10) $Q^{\prime }=\theta \left(S+E_{S}+E_{M}+E_{A}+I_{S}+I_{M}+I_{A}\right)-\left(\mu +\varphi \right)Q$

Adding up the incubation and infected compartments we have the following equivalent system:

(11) $S^{\prime }=\mu -fS-\left(\mu +\theta \right)S+q_{1}\varphi Q$
(12) $E^{\prime }=fS-\left(\mu +\;\gamma +\;\theta \right)E$
(13) $I^{\prime }=\gamma E-\left(\mu +\eta +\theta \right)I+\left(1-q_{1}\right)\varphi Q$
(14) $R^{\prime }=\eta I-\mu R$
(15) $B^{\prime }=h+h^{0}I-dB$
(16) $Q^{\prime }=\theta \left(S+E+I\right)-\left(\mu +\varphi \right)Q$

We use the above model to simulate the evolution of the outbreak for 30 days and assumed that chemoprophylaxis was given *a* days after the start of *Leptospira* transmission. The latter was done by modifying *θ* to H(t − a)*θ* where H is the Heaviside function. Table 1 shows the baseline parameter values for these simulations. For some parameters in which the baseline value was not found in the literature review we included a default or predefined parameter commonly used in infectious disease modeling. Outcomes evaluated included total number of leptospirosis cases and number of cases in categories of illness severity. A sensitivity analysis was carried out to evaluate the impact of key parameters, in particular the rate of chemoprophylaxis administration (0.01–0.5) and the duration of chemoprophylaxis (7–28 days) on the number of leptospirosis cases.

## 3. Results

### 3.1. Literature Review on Chemoprophylaxis Use for Leptospirosis

**Figure 2:**
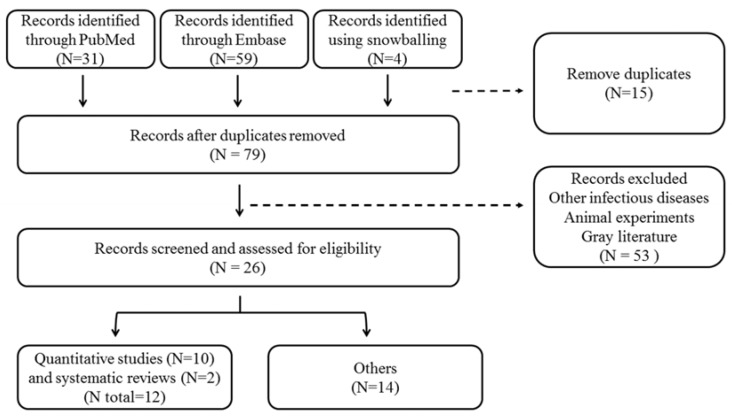
Flow chart for the literature review on chemoprophylaxis use for leptospirosis.

Twenty-six peer-reviewed publications satisfied our selection criteria (Figure 2) and were submitted to a full-text analysis, including 10 quantitative studies [37,38,46,47,48,49,50,51,52,53], two systematic reviews [39,54] and 14 articles of other types [35,36,44,45,55,56,57,58,59,60,61,62,63,64] (Table 2 and Table A2).

The identified quantitative studies included randomized and non-randomized controlled trials, cohort and case-control studies, as well as theoretical modeling with decision trees. Both systematic reviews and almost all quantitative studies (8 of 10) assessed prophylactic effect of oral doxycycline with small variation on its duration and dosage. Pre-exposure prophylaxis for populations in endemic areas or risk groups was assessed in five studies; the results showed a significant protective effect for morbidity and mortality, but unclear effectiveness for infection [37,47,48,51,53]. Galloway et al. used a decision tree model to evaluate the effectiveness and cost effectiveness of early antibiotic therapy in patients with leptospirosis and concluded that prophylactic treatment reduced the severity and mortality of illness with cost savings [50].

Post-exposure prophylaxis was assessed in four studies, three following floods and one after an outbreak in animals [38,46,49,52]. The Bhardwai et al. study was conducted in India based on a total of 62 laboratory confirmed cases and 253 healthy controls with a reported use of doxycycline as chemoprophylaxis in 42% of cases and 63% of controls [49,65]. The unadjusted association was found to be protective (OR = 0.43, 95% CI = 0.23–0.78). However, when other risk factors such as walking barefoot, contact of wounds with flood waters, and the use of flood waters for various activities were accounted for, the association between chemoprophylaxis and leptospirosis was not statistically significant (Adj OR = 0.77, 95% CI = 0.35–1.69).

Gonsalez et al. reported on a clinical trial pilot study (*n* = 82), double-blinded, randomized, and controlled with a placebo to assess the effectiveness of oral doxycycline (200 mg, single dose) in preventing leptospirosis after high exposure to potentially contaminated water in Brazil [52]. Even though a protective association of doxycycline for confirmed leptospirosis cases (RR = 2.3) was found, the association was not statistically significant. Chusri et al. investigated the protective efficacy of a single dosage of 200 mg doxycycline against leptospiral infection and leptospirosis and associated risk factors among residents exposed to flooding in Thailand [38]. Of 641 participants, 600 received doxycycline while 41 did not. The authors concluded that a single dosage of doxycycline for prophylaxis might be effective for preventing leptospirosis among flood victims with laceration wound (27.6% of them) after recent flood exposure. Although the effectiveness of post-exposure prophylaxis in the literature was inconclusive, the direction of association supported protective effect for morbidity and mortality.

Only one randomized controlled trial evaluated the effectiveness of oral penicillin (500 mg twice per day for a month) [51]. Illangasekera el at. focused on job-related exposure among active farmers in Sri Lanka. Only farmers in the placebo group showed signs of infection and were hospitalized. All other types of studies included in our analysis (Appendix B), evaluated the use of doxycycline as the prophylactic antibiotic treatment for leptospirosis [35,36,44,45,55,56,57,58,59,60,61,62,63,64]. Two studies reported the use of both doxycycline and penicillin [56,64]. However, the majority of these studies described the effectiveness of prophylaxis as uncertain and indicated that stronger evidence is needed from larger clinical trials. Nonetheless, many of them recommended doxycycline for anticipated exposures or short-term visitors to endemic areas, to reduce the severity of symptoms and mortality. Overall, oral doxycycline (200 mg once a week) was the most used prophylactic antibiotic treatment against leptospirosis found in the literature review.

The two identified systematic reviews were from the Cochrane Database of Systematic Reviews and both included a small number of randomized clinical trials [39,54]. Guidugli et al. showed statistically significant reduction of symptomatic and verified leptospirosis incidence in the group with doxycycline [54]. However, Brett-Major et al., who included two pre-exposure and one post-exposure study, showed unclear protective effects [39].

### 3.2. Mathematical Simulation of the Outbreak and Effect of Chemoprophylaxis

**Figure 3:**
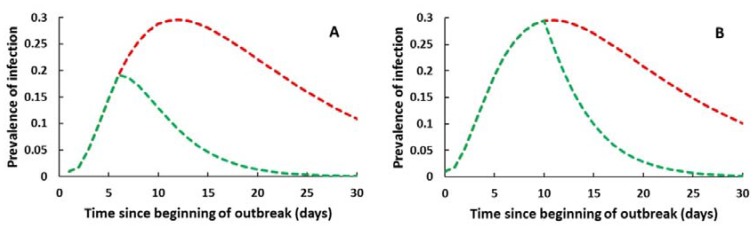
Effect of chemoprophylaxis on the total number of leptospirosis cases. Prevalence of infection (total cases) without (red line) and with (green line) chemoprophylaxis initiated at day 5 (**A**) and at day 10 (**B**) at a rate (1/*θ*) of 0.2.

**Figure 4:**
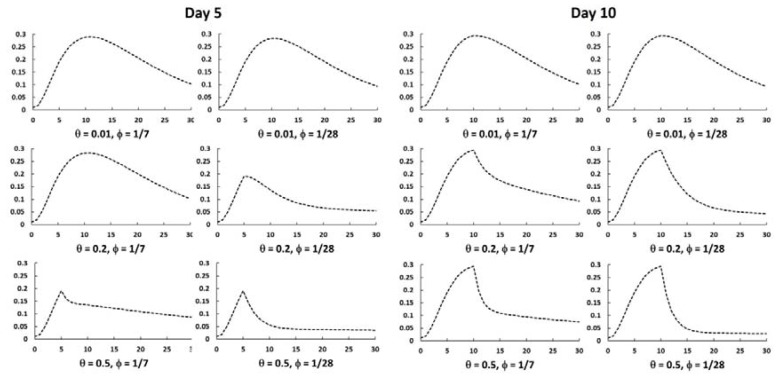
Impact of chemoprophylaxis initiated at day 5 and at day 10, at varying rates (*θ*) and durations (∅), on total number of leptospirosis cases.

Model assumptions under no intervention resulted in a leptospirosis outbreak which quickly peaked at day 12 with 29.4% infection prevalence (Figure 3). Both the timing and the extent of prophylaxis administration influenced the outcome. Early administration resulted in more cases prevented (84% reduction when administered at day 5 vs. 77% at day 10, *θ* = 0.2) (Table 3). However, a delayed application but with higher rate *θ* = 0.5 improved the proportion of cases prevented to 88%. Slower administration rates of 0.01 or 0.05 prevented up 47% of cases. Evaluation of administration rates and duration revealed that the outbreak decreased at a slower pace with a shorter duration of chemoprophylaxis. The largest reduction in the outbreak impact resulted with a fast administration starting at day 5 and continuing for 28 days (Figure 4). Baseline conditions assumed cases presented as 50% symptomatic (20% severe and 30% mild) and 50% asymptomatic. There is limited evidence on the impact of chemoprophylaxis on changes in illness severity; however, assuming chemoprophylaxis (at day 5) reduced occurrence of severe cases to *p*1 = 0 while maintaining 50% symptomatic cases (*p*2 = 50%), the overall illness distribution among all leptospirosis cases would change to 2% severe cases, 48% mild cases, and 50% asymptomatic.

## 4. Discussion

This study was conducted to support decision-making in the health sector in the possible event of a leptospirosis outbreak after a flood in a high-risk area. Prophylactic antibiotic treatment has been used by some countries in the Americas Region following epidemic events after heavy rains, particularly in rural areas when access to the health services is difficult [35,36,37,38]. Although leptospirosis outbreaks do not always occur after a flood or heavy rain, and there are many other factors underpinning the occurrence of leptospirosis, health authorities in many countries recognize the need to address risks and to be prepared to respond to possible outbreaks, particularly during the rainy season.

Based on our literature review, oral doxycycline (200 mg oral/week) was the most commonly used prophylactic antibiotic treatment against leptospirosis. Among the studies that evaluated post-exposure prophylaxis, which would be the case in a flood-associated outbreak scenario, the direction of the association suggested a protective effect for reduced leptospirosis morbidity and mortality; however, evidence is inconclusive. Furthermore, it is important to emphasize that the systematic reviews and other publications recommend that stronger evidence is still needed to fully inform mass chemoprophylaxis interventions.

Most of the studies found in the literature review were from South-East Asian countries, the world sub-region with the highest burden of leptospirosis [22]. Two studies were conducted in the Americas and none in Africa, regions where the burden of leptospirosis is also estimated to be high [22]. Recent publications focusing leptospirosis in Africa demonstrate the importance of this disease in humans and in animals [66] and the need for more information [67].

The available information used in the compartmental SEIR model of this study was limited. Even though *Leptospira* can survive in water and soil and contribute to human and animal infections, it has been difficult to measure the epidemiological parameters needed for modeling purposes. With the expansion of the polymerase chain reaction (PCR) technique, an increasing number of studies are reporting detection and quantification of *Leptospira* in environmental samples. For example, various studies in small communities in the Americas have reported around 20% of environmental samples test positive for pathogenic *Leptospira* [68,69]. However, data measuring contact rates, contamination levels and infective dose, in particular under flood conditions, are lacking. The epidemic curve simulated here was based on a significant level of exposure in a short period of time and a four-week incubation period, which has been reported in previous studies or unpublished data presented by country authorities [70,71,72,73].

The basic mathematical modeling exercise underscores the impact of chemoprophylaxis timing and administration rate as important factors determining the change in the course of the outbreak, and therefore, the potential benefit of the intervention. The example simulated an explosive outbreak, which could result from a large number of people being exposed to *Leptospira*-contaminated flood water, and where an early and fast chemoprophylaxis program would result in the greatest reduction in cases. In other settings, for which the inherently heterogeneous nature of *Leptospira* exposure drives infection risk [74], the effect of these parameters is expected to differ. The model assumed protection from infection while under chemoprophylaxis and evaluated scenarios for changes in illness severity in infected individuals. However, the severity of a leptospirosis case could also be related to other factors, such as the *Leptospira* serovar, the patient humoral immune response and age [21,75,76]. However, empirical data to inform model parametrization are limited.

The public health implications of leptospirosis outbreaks and the associated emergency responses require multidisciplinary research and multi-sectorial collaboration to fill knowledge gaps and to develop future models that capture the necessary complexity while assessing benefits and any potential detrimental effects [77,78]. More information is needed and it will be interesting to examine together with countries’ authorities and scientists the evolution of outbreaks under the realistic conditions after floods; however, during outbreaks the priority is saving lives.

According to the World Health Organization and the International Leptospirosis Society (WHO/ILS) guidelines, chemoprophylaxis is not the sole approach to prevent leptospirosis [20]. A list of the recommended control strategies and interventions based on the WHO/ILS guidelines is presented in Supplementary file S1. Early case detection and strong diagnostic capabilities can reduce the number of severe cases and deaths. In the event of an outbreak, heath authorities need to be prepared to support clinical and laboratory diagnosis, have guidelines for patient treatment, available antibiotics and more specialized care, if needed. Educational campaigns to health workers, first responders and the community at risk are also important components for disease control during an outbreak. Health workers should be educated to recognize the disease and understand suitable treatments. First responders need to be instructed about preventive measures and use of personal protection equipment, such as boots and gloves. In addition, the community should be informed of the clinical signs of leptospirosis and the risk of exposure and prevention strategies, which may include boiling drinking water, avoiding contact with contaminated water and using protective clothing [20]. Outbreak response also requires working in collaboration with other sectors, especially civil defense, disaster responders and other groups that work with health emergencies. The agriculture and environmental sectors, as well as research institutions and universities need to collaborate to achieve better results to predict, prevent, detect and respond to leptospirosis outbreaks in risk areas. Tropical and subtropical countries should include leptospirosis in their emergency disaster plans, as done by Brazil [79]. The Global Leptospirosis Environmental Action Network (GLEAN) [80], a group comprised of multi-disciplinary and multi-sectorial leptospirosis experts from international organizations, research institutions, universities and foundations, acknowledges that additional research is needed to provide evidence-based recommendations for leptospirosis control during an outbreak, but a preliminary list of recommendations has been outlined [81] (Supplementary file S2). The prevention and control strategies of this public health threat need to be addressed using the One Health concept [82]. Even though major efforts have been made by scientists and country authorities in understanding the disease and establishing surveillance and control programs, leptospirosis remains a neglected disease facing unawareness, significant underdiagnoses and a lack of effective tools for prevention and control actions, such as human vaccines and accurate, inexpensive rapid diagnostic tests [83]. One of the reasons for this could be the complexity of transmission cycles involving a range of animal carriers, and several species and serovars as etiological agents, as well as different environmental factors. This complexity poses a biological, an environmental and also a scientific challenge that could be better addressed by using the “One Health” approach for the ultimate goal of saving lives [82]. In this comprehensive approach, climate change and extreme weather events such as floods need to be included. The scientific community, health and others sector authorities and international organizations need to work together to provide the necessary data to support informed decision-making when addressing the risks of leptospirosis outbreaks. There is also a need to advance in developing other tools to prevent leptospirosis cases, such as vaccines, to be used for high risk groups or areas.

## 5. Conclusions

The frequency and intensity of extreme weather events, especially heavy rainfall and floods are projected to increase, and this increase is likely to enhance the risk of leptospirosis outbreaks. In a systematic review of the published literature on mass chemoprophylaxis to reduce the health impact of leptospirosis, we found that oral doxycycline was the most used antibiotic. Although the evidence for effectiveness of post-exposure prophylaxis in the literature is inconclusive, the observed direction of association supported a protective effect for morbidity and mortality. However, additional research is needed in order to understand the direct benefit of chemoprophylaxis on leptospirosis infection and illness and to identify factors influencing benefits and risks under different settings.

## Figures and Tables

**Table 1:** Model parameter description and baseline values.

Parameter	Description	Baseline	References
*β*	Per capita contact rate (1/day)	1.42	Set parameter (default)
*k*	Half-saturation density	0.5	Set parameter (default)
*µ*	Mortality rate by other causes	0	Set parameter (default)
*h*	Leptospire recruitment rate into environment (1/day)	0.002	Set parameter (default)
*d*	Leptospire clearance rate in environment (1/day)	0.2	[42]
*γ*	Incubation period (days)	10	[20]
*η*	Infectious period (days)	7	[43]
*p*	Probability of severe leptospirosis given infection	0.2	Based on Bharti et al. for icteric and other severe forms [44]
*q*	Probability of mild leptospirosis given infection	0.3	Based on Bharti et al. and Faucher et al. 80% of infections are mild or asymptomatic [44,45]
*θ*	Chemoprophylaxis administration rate (1/day)	0.2	Set parameter (default)
*φ*	Chemoprophylaxis duration (days)	14	[46]
*p_1_*	Probability of developing severe leptospirosis after chemoprophylaxis	0.1	Set parameter. 50% lower than before chemoprophylaxis
*p_2_*	Probability of developing mild leptospirosis after chemoprophylaxis	0.15	Set parameter. 50% lower than before chemoprophylaxis
*q_1_*	Probability of not being infected after chemoprophylaxis	0.3	Based on Seghal et al. but with a lower protective effect [47]

**Table 2:** Summary of quantitative studies and systematic reviews on the effectiveness of chemoprophylaxis for leptospirosis.

Author [Ref]	Year	Country	Antibiotics	Target Population	Situation	Administration	Effectiveness of Treatment
Chusri [38]	2014	Thailand	Doxycycline (200 mg single dose)	Local residents	Flooding	Post exposure	Protective efficacy for leptospiral infection: 92.0% (CI = 81.2%–96.6%) and for leptospirosis: 95.6% (CI = 78.2%–99.3%), among participants with laceration wound. Protective efficacy for leptospiral infection: 89.2% (CI = 63.6%–96.67%), among participants exposed to flood water ≤3 h/day.
Shivaraj [48]	2012	India	Doxycycline (200 mg/week)	Paddy field farmers	Farming	Pre-exposure	Incidence of leptospirosis: nil in the test group and 7.29% in the control group (*p =* 0.017)
Bhardwaj [49]	2010	India	Doxycycline (200 mg/week)	Local residents	Flooding	Post exposure	Univariate analysis: OR = 0.43 (CI = 0.23–0.78). Multivariate analysis: Adj OR = 0.77 (C.I = 0.35–1.69)
Galloway [50]	2009	N/A *	Doxycycline and azithromycin	N/A	N/A	N/A	Prophylaxis with doxycycline compared to no-prophylaxis strategy. Prophylaxis provided cost savings, decreased severity of illness and mortality, and improved health outcomes.
Illangasekera [51]	2008	Sri Lanka	Penicillin (500 mg/day for a month)	Farmers	Farming	Pre-exposure	Of 5 patients hospitalized with fever, 3 tested positive for leptospirosis, all from the placebo group.
Belmaker [46]	2004	Israel	Doxycycline (200 mg/week)	Dairy workers	Animal husbandry	Post exposure	Either with or without chemoprophylaxis, no dairy workers exposed to herds infected with *Leptospira* serovar *Hardjo* showed evidence of seroconversion or disease.
Sejvar [37]	2003	Malaysia	Doxycycline (200 mg/week)	Athletes	Race in risk area	Pre-exposure	Taking doxycycline before or during the race was protective (RR = 0.4, 95% CI = 0.2–1.2).
Sehgal [47]	2000	India	Doxycycline (200 mg/week)	Local residents	Endemic area	Pre-exposure	No statistically difference was observed in the infection rates among the doxycycline and the placebo group. A statistically significant difference was observed in the clinical disease attack rates (3.11 vs. 6.82%) between the two groups.
Gonsalez [52]	1998	Brazil	Doxycycline (200 mg single dose)	Local residents	Flooding	Post exposure	A protective association of doxycycline for confirmed leptospirosis cases (RR = 2.3) and seroconversion only (RR = 2.0) was observed, but it was not statistically significant.
Takafuji [53]	1984	Panama	Doxycycline (200 mg/week)	US Army during deployment	Training	Pre-exposure	95% efficacy. Attack rate of 4.2% in the placebo group compared to an attack rate of 0.2% in the doxycycline group (*p* < 0.001).
Brett-Major [39]	2009	N/A*	Doxycycline	Varied (Meta-analysis)	Varied (Meta-analysis)	Varied (Meta-analysis)	Three randomized clinical trials met the inclusion criteria. Pre-exposure antibiotic prophylaxis with doxycycline may decrease laboratory identified *Leptospira* infection.
Guidugli [54]	2000	N/A*	Doxycycline	Varied (Meta-analysis)	Varied (Meta-analysis)	Varied (Meta-analysis)	Two randomized clinical trials met the inclusion criteria. Doxycycline seems to be an efficient intervention when used in a specific clinical situation, i.e., soldiers who train in endemic areas with high risk of exposure.

* Not applied (N/A) because was a multi countries study.

**Table 3:** Proportion of cases prevented (up to T = 30 days) compared with no chemoprophylaxis as function of chemoprophylaxis rate (*θ*) and day of start of application (*t*_0_).

Time of Administration (t_0_, day)	Chemoprophylaxis Rate (*θ*, 1/day)	Proportion of Cases Prevented
5	0.01	0.12
5	0.05	0.47
5	0.1	0.67
5	0.2	0.84
5	0.5	0.93
10	0.01	0.12
10	0.05	0.43
10	0.1	0.62
10	0.2	0.77
10	0.5	0.88

## Supplementary Materials

The following are available online at www.mdpi.com/1660-4601/14/6/594/s1, Supplementary file S1: Guidelines for Prevention and Control of Leptospirosis, Supplementary file S2: GLEAN Recommendations for Outbreak Control.

## Appendix A

**Table A1:** Number of natural disasters by type and subtype, Central America, 2000–2016.

Type	Subtype	2000	2001	2002	2003	2004	2005	2006	2007	2008	2009	2010	2011	2012	2013	2014	2015	2016	Total
Drought	Drought	2	4	2	0	1	0	0	0	0	3	0	1	2	1	3	3	0	22
Earthquake	Ground movement	2	3	0	4	1	1	1	1	0	2	1	2	3	1	4	0	0	26
Epidemic	-	0	0	0	1	0	0	0	0	0	0	0	0	0	0	0	0	0	1
	Bacterial disease	0	0	1	0	0	0	0	0	0	0	1	0	0	0	0	0	0	2
	Viral disease	1	0	3	0	0	0	0	0	0	4	1	0	0	4	1	1	0	15
Extreme temperature	Cold wave	1	1	1	1	1	0	3	0	0	0	0	2	0	0	0	0	0	10
	Severe winter conditions	0	0	0	0	0	0	0	0	0	0	0	0	0	0	1	0	0	1
Flood	-	3	2	1	1	0	1	0	1	0	0	0	0	0	0	0	2	4	15
	Coastal flood	0	0	1	1	0	0	0	1	0	0	0	0	0	0	0	0	0	3
	Flash flood	1	2	1	1	2	0	0	0	2	0	0	0	0	0	0	0	1	10
	Riverine flood	4	0	9	4	5	9	4	9	12	9	13	12	4	2	5	6	1	108
Landslide	Landslide	1	0	1	2	1	2	2	1	2	0	3	1	0	0	0	1	1	18
Mass movement (dry)	Landslide	0	0	0	0	0	0	0	0	0	1	0	0	0	0	0	0	0	1
Storm	-	0	0	0	0	1	0	0	0	0	0	0	0	0	0	0	0	0	1
	Convective storm	2	0	0	0	0	0	0	0	0	0	0	0	0	0	0	3	0	5
	Tropical cyclone	4	10	6	2	0	16	3	9	6	4	15	6	2	4	7	1	8	103
Volcanic activity	Ash fall	2	0	2	0	0	1	0	0	0	0	1	0	1	1	0	1	0	9
Wildfire	-	0	0	0	0	0	0	1	0	0	0	0	0	0	0	0	0	0	1
	Forest fire	1	0	1	2	0	0	0	0	0	0	0	0	0	0	0	0	0	4
	Land fire	0	0	0	0	0	0	0	0	0	0	0	1	0	0	0	0	0	1
Total		24	22	29	19	12	30	14	22	22	23	35	25	12	13	21	18	15	356

Source: International Disaster Database from the Centre for Research on the Epidemiology of Disasters.

## Appendix B

**Table A2:** Other publications on the effectiveness of chemoprophylaxis for leptospirosis.

Author	Year	Journal	Type of the Study	Prophylactic Antibiotic	Recommendations/Conclusions
Devishree [55]	2015	*J. Pharm. Sci. Res.*	Review	Doxycycline	Pre-exposure: doxycycline (200 mg/week). Post exposure prophylaxis with doxycycline based on the degree of exposure (low, moderate, high)
Charan [56]	2012	*Nat. J. Physiol. Pharm. Pharmacol.*	Review	Doxycycline and penicillin	The role of antibiotics in chemoprophylaxis of leptospirosis is uncertain due to lack of large scale trials. More evidence based studies are required to generate evidence for antibiotics being used as chemoprophylaxis.
Dechet [35]	2012	*PloS ONE*	Chemoprophylaxis campaign description	Doxycycline	The effectiveness of the massive chemoprophylaxis campaign was inconclusive.
McBride [57]	2010	*Pharmaceuticals*	Review	Doxycycline	May only act to reduce clinical illness rather than infection. May cause nausea and vomiting
Cruz [58]	2009	*Ethn. Dis.*	Review	Doxycycline	When high-risk and short-term exposure to leptospira is anticipated, chemoprophylaxis is effective. Doxycycline prophylaxis does not prevent leptospiral infection in endemic areas, but has a protective effect in reducing morbidity and mortality during outbreaks.
Pavli [59]	2008	*J. Travel Med.*	Review	Doxycycline	Pre-exposure doxycycline chemoprophylaxis should be considered for adventure travelers, athletes, and military recruits involved in high-risk activities in endemic areas.
Christopher [60]	2005	*Milit. Med.*	Review	Doxycycline	Dr. E.T. Takafuji and colleagues at the Walter Reed Army Institute of Research demonstrated that prophylaxis using doxycycline conferred a 95% risk reduction.
Edwards [61]	2004	*Exp. Rev. Anti Infect. Ther.*	Review	Doxycycline	Short-term chemoprophylaxis with doxycycline in healthy young adults is effective but larger studies are required to demonstrate effectiveness in other ages and resident populations.
Faucher [45]	2004	*Exp. Opin. Pharmacother.*	Review	Doxycycline	The efficacy of pre-exposure doxycycline has been established by two randomized studies performed in different epidemiological environments. However, the efficacy of doxycycline in post exposure prophylaxis is not firmly established.
Levett [62]	2004	*Clin. Appl. Immunol. Rev.*	Review	Doxycycline	Doxycycline may be considered for chemoprophylaxis if high-risk exposures are anticipated.
Bharti [44]	2003	*Lancet Infect. Dis.*	Review	Doxycycline	Chemoprophylaxis may be impractical to administer in highly endemic areas, but is likely to be useful for adventure travelers and military personnel who visit endemic areas, and also in accidental laboratory infection.
Lo Re III [63]	2003	*Am. Fam. Physic.*	Clinical recommendation	Doxycycline	Doxycycline is an effective prophylaxis for travelers to endemic areas who have a high risk of exposure.
Haake [36]	2002	*Clin. Infect. Dis.*	Case report	Doxycycline	The benefits of doxycycline prophylaxis must be weighed against the potential adverse side effects of prophylaxis. Doxycycline may be used as a chemoprophylaxis strategy against both malaria and leptospirosis for patients who anticipate having a relatively high level of exposure. Clinical trials are needed to validate antibiotics with longer serum half-lives, such as azithromycin.
Gilks [64]	1988	*Postgrad. Med. J.*	Case report	Doxycycline and penicillin	Doxycycline, 100 mg twice weekly, over a period of 6 weeks provides a rational regimen for post-exposure prophylaxis, which takes into account the possibility of prolonged incubation.
