# Supplementary material for: The Use of Chemoprophylaxis after Floods to Reduce the Occurrence and Impact of Leptospirosis Outbreaks
Source: Int J Environ Res Public Health. 2017 Jun 3;14(6):594. doi: 10.3390/ijerph14060594 (PMC5486280; doi:10.3390/ijerph14060594)
Supplement: Supplementary file 1 [file ijerph-14-00594-s001.pdf]

Supplementary File S1—Guidelines for Prevention and Control of Leptospirosis

Guidelines based on the World Health Organization/International Leptospirosis Society publication titled *Human leptospirosis: guidance for diagnosis, surveillance and control*

Complete reference:

World Health Organization. Human leptospirosis: guidance for diagnosis, surveillance and control. Geneva, Switzerland: WHO; 2003. 1-109 p.

Prevention and control

The control of leptospirosis is complex and depends on the local conditions, due to the large number of serovars and infection sources and the wide differences in transmission conditions. It can be achieved by controlling or reducing infection in animal reservoir populations, such as dogs or livestock. Control of wild animals may be difficult.

Preventive measures must be based on risk groups and local epidemiological factors. Prevention and control should be targeted at: (a) the infection source; (b) the route of transmission between the infection source and the human host; or (c) infection or disease in the human host.

Control of infection source

Control measures should target the local animal species that are the infection sources in a particular area.

These measures include:

- Reducing certain animal reservoir populations, e.g. rats;
- Separating animal reservoirs from human habitations by using fences and/or screens;
- Immunizing dogs and livestock;
- Removing litter and keeping areas around human habitations clean;
- Encouraging people not to leave food around, especially in recreational areas where rats may be present.
- Raising awareness of the general population, risk groups, human and animal health professionals.

Other examples can be found in the Annex 3 of the referenced publication.

Interruption of transmission

In order to interrupt the transmission cycle, it is important to understand the risk factors for human infection and, if possible, the source of infection. Avoiding contact with animal urine, infected animals or an infected environment reduce the risk of infection. In situations where exposure is likely to occur, e.g. occupational or recreational settings, the use of protective clothing and waterproof dressings to cover wounds is recommended to reduce the chance of infection.

Refer to the Annex 3 of the referenced publication for a more complete list.

Human protection

Knowing how, where and when humans may become infected in a particular area is fundamental to develop preventive measures. Raising awareness of leptospirosis among the general population, risk groups and health care providers can lead to faster detection and treatment of the disease.

Human Immunization

Information on human vaccines is limited, but they seem to provide a certain degree of protection. However, immune responses are mostly serovar-specific. Areas where many *Leptospira* serovars occur, vaccines must consist of a mixture of the different serovars circulating locally. Some countries, including Cuba, France and China, have human vaccines available for high risk groups.

In areas where more serious forms of leptospirosis occur and where access to medical services is limited or delay in receiving treatment is likely, vaccines are very important. However, protection is of relatively short duration, and boosting at regular intervals is necessary to maintain protective titres of antibodies. Vaccines may also produce side effects, such as pain at the injection site, and fever.

Animal immunization

Animals can be immunized with vaccines consisting of suspensions of killed leptospires. However, like human vaccines, protection is largely serovar-specific. Even though animal immunization may prevent the development of the disease, it does not always prevent the development of renal carriage.

Decontamination

Leptospira is sensitive to environmental influences and can be killed by disinfectants or desiccation in small areas, such as floors. In large natural environments, such as lakes or rivers, it is not possible to be decontaminated. *Leptospira* can survive in the environment for a long period of time, especially under suitable conditions, e.g. in moist or volcanic origin soil or surface water with a neutral or slightly alkaline pH.

Supplementary File S2 – GLEAN Recommendations for Outbreak Control

April 2013

Global Leptospirosis Environmental Action Network (GLEAN)*

Preliminary GLEAN recommendations for the control of disaster related leptospirosis outbreaks

While it was acknowledged that additional research needs to be conducted in order to provide evidence-based recommendations, GLEAN experts developed a preliminary set of recommendations in order to advise public health leaders if an outbreak occurs prior to formal recommendations being developed.

- Laboratory specimen collection:
    - A case definition should be used in accordance with WHO local possibilities of diagnostic (probable/suspect/confirmed case)
    - As many laboratory specimens as possible should be collected
    - Screening should be done for all suspect cases
    - High quality specimens using serum (if possible whole blood) and sent to appropriate reference laboratory for confirmation
        - MAT, PCR
            - Identification of agent
    - In parallel to reference laboratory confirmation, perform in country tests
        - ELISA or commercial RDT
- Empiric treatment of probable cases:

    - Use empiric treatment for probable cases to reduce morbidity and mortality related to leptospirosis
- Individual prevention:
    - Use barrier protection if there is a potential to come into contact with contaminated materials. If individual exposure occurs when cleaning up after disasters, immediately clean the affected body areas.
    - Treated water should be provided according to water treatment guidelines
- Community prevention:
    - Mass Chemoprophylaxis:
        - There is no current evidence that mass chemoprophylaxis given to the whole population reduces morbidity or mortality
        - There is evidence that pre-exposure chemoprophylaxis decreases morbidity in controlled target populations with individuals of high risk such as military workers, disaster relief workers, sewage and sanitation workers
    - Decontamination of water:
        - There is no indication for mass decontamination of water (based on current evidence).
- Vaccination (human and animals):
    - Based on current evidence, mass immunization with current vaccines does not impact the course of an outbreak.
- Risk communication:
    - Follow a risk communication plan that targets the public sector as well as the health service sector
    - Communicate early to improve awareness among the community and health care providers
    - Communicate risk in a professional and non-exaggerated format
- Rodent control:
    - Rodent control should not be implemented once an outbreak has occurred as it is too late to have a significant impact on human disease transmission

* https://sites.google.com/site/gleanlepto/home
